# Adjuvant Selection for Influenza and RSV Prefusion Subunit Vaccines

**DOI:** 10.3390/vaccines9020071

**Published:** 2021-01-20

**Authors:** Ariel Isaacs, Zheyi Li, Stacey T. M. Cheung, Danushka K. Wijesundara, Christopher L. D. McMillan, Naphak Modhiran, Paul R. Young, Charani Ranasinghe, Daniel Watterson, Keith J. Chappell

**Affiliations:** 1 School of Chemistry and Molecular Biosciences, The University of Queensland, St Lucia, QLD 4072, Australia; a.isaacs@uq.edu.au (A.I.); s.cheung@uq.edu.au (S.T.M.C.); c.mcmillan1@uq.edu.au (C.L.D.M.); n.modhiran@uq.edu.au (N.M.); p.young@uq.edu.au (P.R.Y.); d.watterson@uq.edu.au (D.W.); 2 Department of Immunology and Infectious Disease, The John Curtin School of Medical Research, The Australian National University, Canberra, ACT 2601, Australia; zheyi.li@anu.edu.au (Z.L.); charani.ranasinghe@anu.edu.au (C.R.); 3 The Australian Institute for Biotechnology and Nanotechnology, The University of Queensland, St Lucia, QLD 4072, Australia; d.wijesundara@uq.edu.au; 4 Australian Infectious Disease Research Centre, The University of Queensland, St Lucia, QLD 4072, Australia

**Keywords:** adjuvant, vaccine, influenza, RSV, microbiology, virology

## Abstract

Subunit vaccines exhibit favorable safety and immunogenicity profiles and can be designed to mimic native antigen structures. However, pairing with an appropriate adjuvant is imperative in order to elicit effective humoral and cellular immune responses. In this study, we aimed to determine an optimal adjuvant pairing with the prefusion form of influenza haemagglutinin (HA) or respiratory syncytial virus (RSV) fusion (F) subunit vaccines in BALB/c mice in order to inform future subunit vaccine adjuvant selection. We tested a panel of adjuvants, including aluminum hydroxide (alhydrogel), QS21, Addavax, Addavax with QS21 (AdQS21), and Army Liposome Formulation 55 with monophosphoryl lipid A and QS21 (ALF55). We found that all adjuvants elicited robust humoral responses in comparison to placebo, with the induction of potent neutralizing antibodies observed in all adjuvanted groups against influenza and in AdQS21, alhydrogel, and ALF55 against RSV. Upon HA vaccination, we observed that none of the adjuvants were able to significantly increase the frequency of CD4^+^ and CD8^+^ IFN-γ^+^ cells when compared to unadjuvanted antigen. The varying responses to antigens with each adjuvant highlights that those adjuvants most suited for pairing purposes can vary depending on the antigen used and/or the desired immune response. We therefore suggest that an adjuvant trial for different subunit vaccines in development would likely be necessary in preclinical studies.

## 1. Introduction

Since the use of vaccination to eradicate smallpox, vaccines have emerged as a powerful tool for prevention and control of existing and emerging pathogens. Numerous vaccines have rapidly emerged amidst the COVID-19 pandemic for clinical validation, allowing the comparison of inactivated, recombinant viral, mRNA, and subunit vaccine platforms for safety and immunogenicity in completed Phase I trials that have been published following peer review [1]. From this analysis, subunit vaccines exhibited the most favorable safety and immunogenicity profile [1]. Subunit vaccines possess a high safety profile due to the lack of viral replication, no risk of incomplete inactivation, no live virus components, and minimal side effects [2,3]. Furthermore, subunit vaccines can be rationally designed to a specific conformation to mimic the pathogen structure and elicit a potent neutralizing antibody response. 

Adjuvants are pivotal in enhancing the immune responses elicited against protein subunits. There are numerous good manufacturing practice (GMP)-compatible and scalable adjuvants that can be co-delivered with subunit vaccines to tailor type 1 and/or type 2 T- and B-cell responses that represent immune correlates of natural recovery or resolution of infection [4,5,6,7]. Aluminum salts (hereafter referred to as alum) are the most widely used adjuvants in vaccines, with a strong safety profile shown following hundreds of millions of doses administered in humans [8,9,10]. Alum is thought to act through a combination of local inflammation, the depot effect, and increased uptake of antigen by antigen-presenting cells (APCs), leading to a T-helper 2 (Th2)-biased response [9,10,11]. Moreover, it is known that alum is a poor inducer of T-helper 1 (Th1) response as it does not promote the induction of IL-12 production [12,13]. In contrast, monophosphoryl lipid A (MPLA), a bacterial derivative and toll-like receptor (TLR) 4 agonist, skews the immune response towards a Th1-biased response [14,15,16]. When used in conjunction with alum as part of GlaxoSmithKline’s (GSK) AS04, this adjuvant system is able to improve both humoral and cellular immune responses. As such, AS04 is part of vaccines against hepatitis B virus, human papillomavirus, and herpes simplex virus [14,17]. GSK has also developed additional proprietary adjuvant systems, namely AS01, AS02, and AS03. AS01, a combination of liposomes, MPLA, and saponin QS21, induces robust Th1 immune responses, enhances antigen presentation to APCs, and induces a high antibody titre [17,18,19]. AS02, a squalene emulsion with MPLA and QS21, induces Th1-biased response and a high antibody titre [17,20]. AS03, containing DL-α-tocopherol, squalene, and polysorbate 80, enhances vaccine-generated immune responses, particularly long-term antibody levels, through induction of CD4^+^ T-cell responses [21]. An additional squalene oil-in-water emulsion that has had success as an adjuvant in human vaccines is MF59. MF59 enhances immune responses through a range of immune-stimulatory effects, including increasing the recruitment of immune cells to the injection site through induction of chemokines and enhancing the priming of naïve T-cells by increasing dendritic cell maturation [22,23,24]. MF59’s success as a safe and potent adjuvant has led to the development of Addavax, an MF59-like squalene oil-in-water emulsion. 

We have previously developed subunit vaccine candidates for both influenza and respiratory syncytial virus (RSV) [25] based on influenza haemagglutinin (HA) and RSV fusion glycoprotein (F). Current influenza vaccines are either attenuated, inactivated, and detergent-split or subunit vaccines and provide little cross-protection to other subtypes [26,27,28]. Because these vaccines primarily serve to boost preexisting immunity in primed populations, they are typically unadjuvanted and semi-purified, and therefore result in reduced immunogenicity in unprimed populations, such as young children [29,30]. Furthermore, egg-grown vaccines are only reliable when vaccine strains are matched with circulating strains and therefore require frequent reformulation [26,27,28]. For RSV, there is currently no licensed vaccine, with development hampered by the failure of an alum-adjuvanted formalin-inactivated RSV vaccine in the 1960s [31,32]. Currently, there are several candidates in clinical trials, consisting of AS01B-adjuvanted RSV F subunit vaccine [33], several alum-adjuvanted or unadjuvanted RSV F subunits [34,35], and several unadjuvanted live-attenuated or vectored RSV vaccines [36,37]. Our subunit vaccines for these viruses, currently in preclinical development, make use of a patented technology: a novel trimerization domain, the molecular clamp, that can stabilize viral fusion glycoproteins in their prefusion conformation [25,38,39]. The prefusion conformation allows a tailored and targeted immune response to be elicited against both conserved and highly neutralizing epitopes of the fusion protein used for immunization [40,41]. The clamp stabilization technology is broadly applicable to many viral fusion proteins and has been validated as a platform technology [25,38,39]. As such, we conducted an adjuvant trial to determine optimal adjuvant–antigen pairing for our clamped subunit vaccines using influenza HA and RSV F as model antigens. Here, we selected a panel of adjuvants to test with both clamp-stabilized HA (HA clamp) and clamp-stabilized RSV F (F clamp) that are analogous to what is currently approved or in clinical trials for human use, including aluminum hydroxide (alhydrogel), QS21, Addavax, Addavax with QS21 (AdQS21) and Army Liposome Formulation 55 with MPLA and QS21 (ALF55). 

## 2. Materials and Methods

### 2.1. Antigen Production

Influenza HA clamp and RSV F clamp were produced as previously described [38]. Codon-optimized DNA sequences encoding the ectodomain of HA from A/California/04/2009 (H1N1) (1-510, GenBank: ACP41105.1) or RSV F ectodomain (1-474, GenBank: APW29972.1) with the fusion peptide and peptide 27 deleted (∆106-150) were synthesized by IDT. These sequences were cloned into a mammalian expression vector upstream of a GSG-linked HIV gp41 fusion core trimerization domain using the inFusion cloning method and Stellar competent cells according to the manufacturer’s protocol (TakaraBio, Shiga, Japan). Plasmid DNA sequences encoding HA clamp and F clamp were transfected and expressed using the ExpiCHO-S expression system according to the manufacturer’s protocol (ThermoFisher Scientific, Waltham, MA, USA). In brief, a ratio of 1 µg DNA to 1 mL of cultured ExpiCHO cells at a density of 6 × 10^6^ cells/mL was used for transfection. Seven days after transfection, cell culture supernatant was harvested by centrifugation at 4800× *g* for 30 min at 4 °C prior to filter sterilization (0.22 µm) for subsequent protein purification. Protein was purified from cell culture supernatant using immunoaffinity purification with an in-house-made column embedded with a resin coupled with an anti-clamp monoclonal antibody (HIV1281). Supernatant was applied to the column, washed with high salt PBS (PBS with 400 mM NaCl; pH 7.4), and eluted with a high pH buffer (100 mM glycine, 137 mM NaCl, and 5 mM EDTA; pH 11.5). Eluted fractions were neutralized with a 1:1 v/v ratio of 1 M Tris pH 6.8. Protein was concentrated and buffer exchanged into PBS (Merck Amicon, Burlington, MA, USA), and the protein concentration was quantified using NanoDrop One (ThermoFisher Scientific). 

### 2.2. Adjuvant Formulation and Mouse Immunizations

Adjuvants were formulated at one-tenth of the dose approved for humans. Five micrograms of antigen was formulated with either 5 µg QS21 (Desert King), 50 µg alhydrogel (Brenntag), 1.25 mg Addavax (Invivogen), 5 µg QS21 with 1.25 mg Addavax (AdQS21; Desert King/Invivogen), or 5 µg QS21 with 300 µg liposomes containing 5 µg MPLA (ALF55, Avanti Polar Lipids Inc., Alabaster, AL, USA).

Female BALB/c mice aged 5–8 weeks were sourced from the Australian Resource Centre and housed in individually ventilated, HEPA-filtered cages at the University of Queensland Biological Research facility. The mouse study was conducted in accordance with the University of Queensland Animal Ethics Committee approval (AEC SCMB/558/17). Mice were allowed to acclimatize for 1 week prior to intramuscular vaccination in the hind-leg muscle with the aforementioned adjuvant–antigen formulations, PBS placebo, or antigen alone under anesthesia. Two weeks post prime, half the mice (*n* = 4) were sacrificed for spleen harvest and cardiac puncture. A week later, blood from the remaining mice (*n* = 4) was collected via the tail vein. One day later, a booster immunization was given to the mice as before. Three weeks later, all remaining mice were sacrificed for spleen harvest and cardiac puncture. Serum from all timepoints was harvested by allowing blood to coagulate overnight at 4 °C prior to centrifugation at 10,000× *g* for 10 min at 4 °C. To assess T-cell responses of each mouse, splenocytes were depleted for red blood cells and profiled for cytokine expression as described below. 

### 2.3. T-Cell Cytokine Profiling

Monoclonal antibodies Brilliant Violet 421 anti-mouse CD3 clone 17A2, PE anti-mouse CD4 clone GK1.5, Brilliant Violet 650 anti-mouse CD8 clone 53-6.7, APC anti-mouse IFN-γ clone XMG1.2, PE/Cy7 anti-mouse TNF-α clone MP6-XT22, and Alexa Fluor 488 anti-mouse interleukin-2 (IL-2) clone JES6-5H4 were obtained from BioLegend (San Diego, CA, USA). Intracellular cytokine staining was performed as described previously [42]. Briefly, 2 × 10^6^ splenocytes were plated into each well and stimulated with 5 μg/mL HA peptide pool or DMSO (negative control) for 16 h. Then, 5 h prior to the end of stimulation, 1 μg/mL brefeldin A (BioLegend) was added to each well. Cells were then washed with FACS buffer (2% FBS in sterile DPBS) and surface staining was performed in the dark on ice for 30 min. Cells were fixed with IC FIX buffer (BioLegend), and permeabilized with 1X permeabilization buffer (BioLegend). Intracellular cytokine staining was then performed in the dark on ice for 40 min. The stained samples were then washed twice with FACS buffer and fixed with 0.5% PFA. For each sample, 1 × 10^6^ events were acquired on a BD LSRII Fortessa, and data were analyzed using Flowjo software V10.

### 2.4. ELISAs

Vaccine antigen-specific IgG from mouse serum samples was measured by ELISA. Briefly, 2 µg/mL of antigen was coated on Nunc Maxisorp ELISA plates and incubated at 4 °C overnight. Plates were blocked with 150 µL/well of 5% KPL milk diluent solution concentrate (SeraCare, Milford, MA, USA) in PBS with 0.1% Tween20 (PBST) for 1 h at room temperature. Blocking buffer was removed and replaced with mouse serum samples serially diluted in blocking buffer before incubation at 37 °C for 1 h. Plates were washed thrice in water before adding 50 µL/well of 1:2500 diluted goat anti-mouse HRP-conjugated secondary antibody (Sigma Aldrich, St. Louis, MO, USA) in blocking buffer. Plates were incubated at 37 °C for 1 h and washed as before prior to being developed for five minutes using 50 µL/well of TMB chromogen solution (Life Technologies, Carlsbad, CA, USA). The substrate reactions were stopped by addition of 25 µL/well of 1 M H_2_SO_4_ before reading the plate absorbance at 450 nm. For IgG isotyping, ELISAs were performed as above, except goat anti-mouse isotype-specific secondary (anti-IgG1, -IgG2a, -IgG3, and -IgG2b; Sigma Aldrich) was applied at 1:2500 dilution in blocking buffer for 1 h at 37 °C. This was followed by washing thrice in water, adding HRP-conjugated rabbit anti-goat (Sigma Aldrich) diluted 1:2500 in blocking buffer, and incubating for 1 h at 37 °C. ELISA was then washed and revealed as previously described. Data was fit with one-site specific binding logarithm on Graphpad Prism 9. EC_50_ of sample against vaccine antigen is defined as the reciprocal of the serum dilution required to achieve binding of half of total antigen. 

### 2.5. Plaque Reduction Neutralisation Tests (PRNTs)

To measure the neutralization capacity of the sera, PRNTs were performed. For influenza PRNTs, Nunc flat bottom tissue culture plates were seeded with 6 × 10^4^ of Madin–Darby canine kidney cells (MDCKs) per well in DMEM (Gibco, Gaithersburg, MD, USA) supplemented with 10% heat-inactivated FCS followed by incubation overnight at 37 °C with 5% CO_2_. Mouse serum samples were treated with receptor-destroying enzyme (RDE) (RDE[II]; Denka Seiken Co., Tokyo, Japan) at a ratio of 1 part serum to 3 parts RDE. Samples were incubated overnight at 37 °C before heat inactivation at 56 °C for 30 min. Treated serum samples were serially diluted before incubation for 1 h at 37 °C with 5% CO_2_ with 100 PFU/well of influenza virus (A/Auckland/1/2009 (H1N1)) in DMEM containing 4 µg/mL of TPCK-treated trypsin. Virus-serum mixtures were then added to MDCK cells and incubated for 1 h at 37 °C with 5% CO_2_. Virus–serum mixtures were discarded before adding 100 µL/well of overlay (M199 media (Gibco) consisting of 2% heat-inactivated FCS supplemented with penicillin–streptomycin and 1.5% medium viscosity carboxymethyl cellulose (CMC)) followed by 3 days of incubation at 37 °C with 5% CO_2_. Plates were fixed in 80% acetone/20% PBS for 20 min at −20 °C before blocking in 5% KPL milk diluent solution concentrate (SeraCare) in PBST for 1 h at room temperature. Plates were then stained with 2 µg/mL of anti-HA hFI6V3 monoclonal antibody (mAb) for 1 h at 37 °C. Plates were washed three times in PBST before adding anti-human IR800 (Millennium Science, Mulgrave, Australia) diluted to 1:2500 in blocking buffer. The washing step was repeated, and plaques were revealed by scanning plates on an Odyssey CLX infrared imaging system (LI-COR). Plaques were counted and plotted on Graphpad Prism 9 using a 3-parameter log(inhibitor) vs. response model. 

For RSV PRNTs, Nunc flat bottom tissue culture plates were seeded with 5 × 10^4^ Vero cells/well in OptiMEM (Gibco) supplemented with 3% heat-inactivated FCS and incubated overnight at 37 °C with 5% CO_2_. Heat-inactivated serum samples were serially diluted in serum-free OptiMEM before the addition of RSV A2 virus (produced in Vero76 cells) diluted to 75 PFU/well in OptiMEM. Virus–serum mixtures were incubated for 1 h at 37 °C with 5% CO_2_ before adsorption onto plated cells for an additional hour at 37 °C with 5% CO_2_. Then, 100 µL/well of virus overlay was added to all wells before incubation for 3 days at 37 °C with 5% CO_2_. Plates were fixed and stained with anti-RSV F human motavizumab (1 µg/mL) mAb as described for influenza PRNTs. Plaques were counted and plotted on Graphpad Prism 9 using a 3-parameter log(inhibitor) vs. response model.

### 2.6. Statistics 

Statistical analyses to generate *p*-values were conducted in GraphPad Prism 9 using an ANOVA test adjusted for multiple comparisons using Tukey’s method on T-cell values and transformed log values of EC_50_s and IC_50_s. 

## 3. Results

### 3.1. Humoral Responses

**Figure 1:**
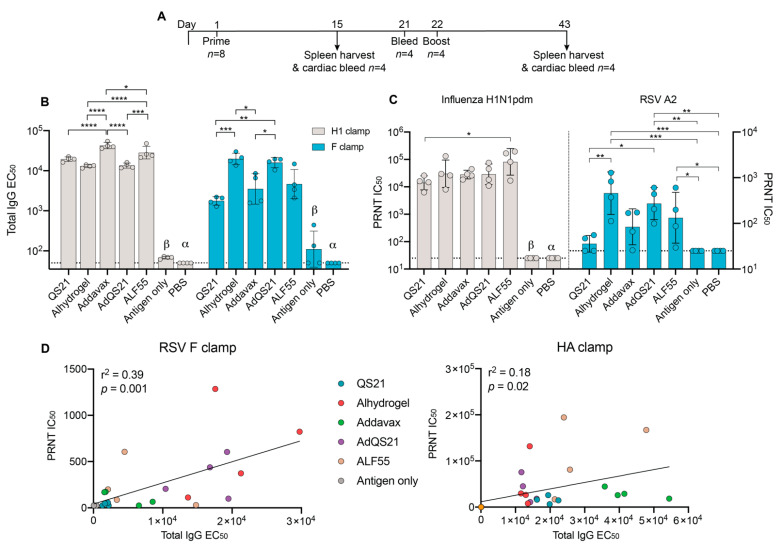
Serum analysis of terminal bleed from mice immunized with clamped antigen with different adjuvants. (**A**) Schematic of immunization regime. (**B**) ELISA EC_50_ of serum against each vaccine antigen on day 43. Dotted line shows limit of detection. (**C**) Serum samples from final cardiac bleed (day 43) tested against homologous H1N1pdm (A/Auckland/1/2009, left) virus or respiratory syncytial virus (RSV) A2 (right) in a plaque reduction neutralization test (PRNT). Data represents geometric mean (*n* = 4) with geometric standard deviation (SD). Dotted line shows limit of detection. *p*-values were calculated using a one-way Tukey’s multiple comparison ANOVA, where * indicates *p* < 0.05, ** *p* < 0.005, *** *p* < 0.0005, and **** *p* < 0.0001; β = *p* < 0.0001 compared to all groups except PBS, and α = *p* < 0.0001 compared to all groups unless otherwise specified. (**D**) Linear regressions of PRNT IC_50_ vs. total IgG EC_50_ from groups vaccinated with RSV fusion (F) clamp (left) and haemagglutinin (HA) clamp (right).

To determine which adjuvant is best suited for our candidate prefusion antigens, an immunization study was performed using a panel of adjuvants analogous to formulations that are currently approved in humans or in clinical development (Figure 1A). After two immunizations of HA clamp, we observed that inclusion of any adjuvant increased humoral immunity significantly beyond placebo (PBS) and antigen only (Figure 1B). Mice immunized with HA clamp with Addavax elicited the highest total IgG in comparison to all groups (Figure 1B). Despite these differences between total IgG, sera from all adjuvanted groups were able to neutralize homologous influenza virus to a similar extent, with ALF55 eliciting the highest neutralization, reaching a statistical significance only in comparison to the QS21 group (Figure 1C). 

In contrast to the HA clamp results, two immunizations with RSV F clamp with either alhydrogel or AdQS21 elicited the highest IgG response, with significant differences observed when comparing alhydrogel to QS21 and Addavax (Figure 1B). Notably, the AdQS21 group elicited significantly higher IgG EC_50_ in comparison to its constituents alone (QS21 and Addavax groups). Following analysis of virus-neutralizing antibody response, all the adjuvanted groups elicited a neutralizing antibody response that was higher than the antigen only and placebo groups in the RSV F clamp study (Figure 1C). However, this was only statistically significant for the alhydrogel, AdQS21, and ALF55 groups (Figure 1C). A general trend was observed for both F clamp and HA clamp immunizations, where higher IgG responses correlated with higher neutralization (Figure 1D; *r^2^* = 0.39, *p* < 0.001 for F clamp; *r^2^* = 0.18, *p* = 0.02 for HA clamp). This was exemplified by RSV F clamp adjuvant groups, where the lower IgG response from the QS21 group was associated with lower neutralization capacity of RSV, and the significantly higher IgG response from the alhydrogel group was associated with higher IC_50_. 

**Figure 2:**
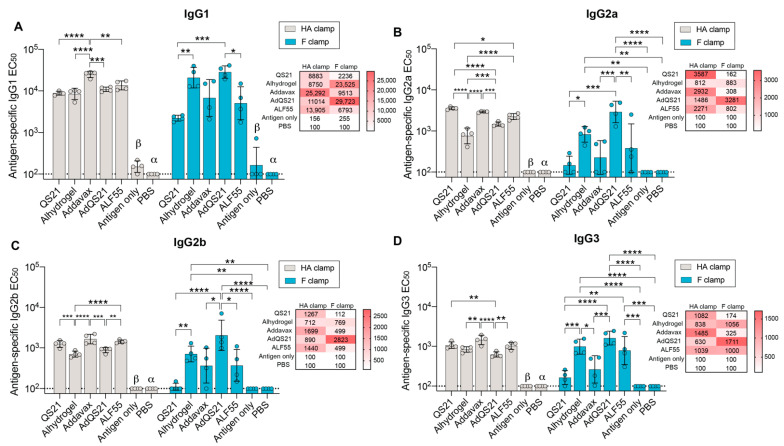
Antibody isotyping of mouse sera post antigen immunization with various adjuvants. Data shown is from the final terminal bleed. Sera from individual mice (*n* = 4) were isotyped by ELISA against the vaccine antigen with secondary antibodies specific for mouse IgG1 (**A**), IgG2a (**B**), IgG2b (**C**), and IgG3 (**D**) with PBS background subtracted. Data represents geometric mean with geometric SD. Dotted line shows limit of detection. A heat map is shown for each isotype, with values representing means of each group. Statistics calculated using a one-way Tukey’s multiple comparison ANOVA, where * indicates a *p* < 0.05, ** *p* < 0.005, *** *p* < 0.0005, and **** *p* < 0.0001; β = *p* < 0.0005 compared to all groups except PBS, and α = *p* < 0.0001 compared to all groups unless otherwise specified.

Both neutralizing and non-neutralizing antibodies have been implicated in protection against influenza and RSV infection [43,44,45,46,47,48]. Non-neutralizing antibodies may offer protection through Fc-mediated effector functions, such as the killing of infected cells by antibody-dependent cellular cytotoxicity (ADCC). ADCC has been implicated in both influenza and RSV infection and vaccination [46,49,50,51,52,53,54]. In the murine model, ADCC is mediated by IgG2a and IgG2b isotypes [55]. As such, to further investigate the quality of response elicited by each adjuvant, sera were isotyped into IgG1, IgG2a, IgG2b, and IgG3 subclasses (Figure 2, Supplementary Materials Table S1). For HA clamp, a robust IgG1 response was observed for all adjuvants (Figure 2A). For RSV F clamp, QS21 had the lowest IgG1 EC_50_ in comparison to all other adjuvanted groups, yet this only reached statistical significance for alhydrogel and AdQS21 (Figure 2A). Additionally, AdQS21 elicited a significantly higher level of IgG1 when compared to ALF55 (Figure 2A). 

IgG2a responses in the mouse model have been previously shown to play a significant role in the outcomes of viral infections, and its production is upregulated by IFN-γ [55]. Generation of IgG2a isotype strengthens pathogen clearance as it is associated with stronger FcγR-mediated activity, allowing for activation of ADCC [55]. In the HA clamp vaccination, alhydrogel was seen to elicit a significantly lower IgG2a response in comparison to all adjuvant groups (Figure 2B). In the RSV F clamp study, AdQS21 elicited an IgG2a response that was significantly higher than its constituents alone (Addavax and QS21) (Figure 2B). Interestingly, this was not recapitulated in the HA clamp study, where QS21 and Addavax elicited significantly higher IgG2a levels in comparison to AdQS21 (Figure 2B). IgG2b stimulates FcγR functions and typically arises early in the humoral response through a T-independent pathway [55]. The IgG2b response between adjuvant groups displayed similarities to the IgG2a pattern for both HA clamp and RSV F clamp (Figure 2C). Here, we observed that immunization with HA clamp with alhydrogel or AdQS21 displayed a decreased IgG2b response. In contrast, these adjuvant groups were the top responders in the RSV F clamp immunizations (Figure 2C). IgG3, involved in complement fixation, was also elicited by all adjuvant groups, with a reduced response for QS21 and Addavax groups with RSV F clamp immunization (Figure 2D). We observed that for the RSV F clamp study, AdQS21 elicited the highest levels of all isotypes.

### 3.2. T-Cell Responses

**Figure 3:**
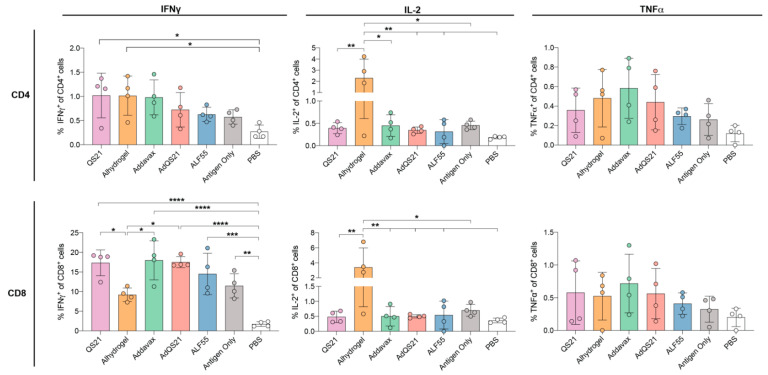
CD4^+^ and CD8^+^ T-cell cytokine profiling of mice immunized with HA clamp alongside various adjuvants. Data shown is from day 43 spleen harvest. Data shown is after subtraction of PBS-stimulated cells. Data represents mean (*n* = 4) with SD. Statistics calculated using a one-way Tukey’s multiple comparison ANOVA, where * indicates a *p* < 0.05, ** *p* < 0.005, *** *p* < 0.0005, and **** *p* < 0.0001.

We next aimed to measure the T-cell responses elicited by immunization with our HA clamp vaccine candidate. Here, CD4^+^ and CD8^+^ T-cells were analyzed for the production of IL-2 and antiviral cytokines IFN-*γ* and tumor necrosis factor (TNF)-*α* to delineate antigen-specific T cell subsets (Figure 3, Supplementary Materials Table S2). In this analysis, we observed that immunization of HA clamp with QS21 or alhydrogel elicited a significantly higher percentage of CD4^+^ IFN-*γ*^+^ cells in comparison to PBS, yet none of the adjuvants significantly increased the frequency of CD4^+^ IFN-*γ*^+^ cells in comparison to antigen alone (Figure 3). Moreover, with the exception of ALF55, alhydrogel elicited a markedly reduced percentage of CD8^+^ IFN-*γ*^+^ cells in comparison to all adjuvants. We also observed that none of the adjuvants were able to significantly increase the frequency of CD8^+^ IFN-*γ*^+^ cells in comparison to antigen alone.

Despite its usage as a parameter to measure vaccine-specific T-cell immunity, induction of IFN-*γ* alone or its magnitude does not correlate with effective protective immunity [42]. However, induction of IL-2 has been shown to be required for the maintenance of memory T-cells [56,57,58]. Strikingly, our results show that upon HA clamp immunization, alhydrogel elicited a significantly elevated CD4^+^ and CD8^+^ IL-2^+^ response in comparison to all other groups tested (Figure 3). Finally, no significant differences in either CD4^+^ and CD8^+^ TNF*α*^+^ cells were observed between any of the adjuvant groups and PBS (Figure 3).

## 4. Discussion

The aim of this study was to determine if a single adjuvant formulation could be used for various clamp-stabilized subunit vaccines. To assess this, we tested the humoral response of the adjuvant panel co-administered with previously developed prefusion-stabilized clamped RSV F or influenza HA. Utilizing this approach, we aimed to elicit prefusion-reactive, conformationally dependent antibodies, which have been shown to be protective for both RSV and influenza [40,41]. Interestingly, we found that formulation of the same adjuvant with different antigens elicited differential humoral responses in terms of total IgG and virus neutralization. This was exemplified by adjuvants like QS21 and Addavax, which elicited high levels of total IgG and neutralized virus efficiently for HA clamp yet did not neutralize RSV above the levels for placebo or antigen alone in the RSV F clamp study (Figure 1). This could in part be a result of different adsorption capacities of antigen to adjuvant, which can be dependent on intrinsic protein properties and can influence stability and biological activity. This is consistent with previous reports of antigen-dependent adsorption on aluminum salts [59,60,61], emulsions [62,63,64], and lipid vesicles [64,65,66]. Furthermore, it has been reported that formulation with certain adjuvants may affect protein structure and stability, which is in part dependent on adjuvant–antigen interactions, with downstream biological consequences in vaccinations [64,67,68]. Moreover, antigens alone may possess immune-stimulatory effects. Indeed, while viral nucleic acids are the prototypical pathogen-associated molecular patterns (PAMPs), which are recognized by pathogen recognition receptors such as TLRs, there have been several reports of viral protein antigens acting as PAMPs. This has been demonstrated for RSV F, influenza HA, and dengue nonstructural protein 1 (NS1), all of which have been shown to activate TLR4 [69,70,71,72], as well as measles virus HA, which has been shown to activate TLR2 [73]. Therefore, we suggest that both absorption capacity and the intrinsic immune-stimulatory effects of antigens may play a role in adjuvant–antigen interactions. This highlights that the adjuvants most suited for pairing purposes can vary depending on the antigen used and/or on the desired immune response.

In the RSV study, we observed a significant positive correlation between antigen-specific total antibody responses and neutralization capacity (Figure 1D). This was exemplified in the alhydrogel, AdQS21, and ALF55 adjuvant groups, where high humoral responses correlated with high neutralization capacities, indicating that these adjuvants elicit potent, favorable humoral responses. Interestingly, the two adjuvant groups that elicited the most potent neutralization (alhydrogel and AdQS21) also elicited the highest amount of the IgG1 isotype (Figure 2A). This is consistent with the primary function of IgG1 in the murine model, which is to neutralize virus through steric hinderance [55]. Other protective functions, such as ADCC, have been deemed important in protection from RSV infection and are mediated by IgG2a and IgG2b isotypes in the murine model [46,52,55]. We found that AdQS21 elicited the highest levels of all isotypes, particularly IgG2a and IgG2b, indicating a well-rounded response (Figure 2). High levels of antigen-specific IgG2a and IgG2b isotype may indicate Fc-mediated effector functions and may contribute to enhanced protection against RSV in vivo [46]. This has been reported in the context of immunotherapy with palivizumab, where it was shown that a human IgG1 isotype (mouse IgG2a homolog) was able to reduce viral titres in lungs of cotton rats to a greater extent in comparison to their human IgG2 counterpart [46,74]. Despite this, the effects of isotypes elicited from immunization on Fc-mediated functions against RSV remains to be investigated.

In comparison to RSV, the positive correlation between antigen-specific antibodies and virus neutralization in the HA study was more subtle but still reached statistical significance (Figure 1D). This trend could be confounded by the presence of non-neutralizing HA stem-specific antibodies, which can be elicited upon vaccination with prefusion HA antigens [75,76]. Non-neutralizing, stem-specific antibodies are typically cross-reactive between influenza subtypes and may offer protection against infection through ADCC [43,76]. We found that all adjuvants were able to elicit IgG2a and IgG2b antibodies above antigen only vaccination, with QS21 eliciting the highest level of IgG2a (Figure 2). This is a favorable response as high levels of HA-specific IgG2a could contribute to increased protection, as previously reported in murine influenza protection studies [43,77,78].

In the HA clamp study, we observed that none of the adjuvants were able to improve production of vaccine-specific IFN-γ and TNF-α by CD4^+^ and CD8^+^ T-cells in comparison to antigen alone; however, longer splenocyte peptide stimulation may result in more effective recall. This was consistent with what has been observed with other adjuvanted viral subunit vaccines [79,80]. However, alhydrogel was able to elicit relatively elevated vaccine-specific IL-2 in both CD4^+^ and CD8^+^ T cells subsets. While IL-2 has no direct antiviral activity, it is known to help maintain expansion and proliferation of antigen-specific T-cells, ensuring longevity and the ability of these cells to establish immunological memory [81,82]. This has been observed in HIV-specific CD4^+^ T-cells [82,83,84], specifically those associated with mucosal protection [56]. More pertinently, IL-2 has also been implicated in the expansion of CD8^+^ T-cells upon influenza infection and as such may be an important contributor to cellular immunity against infection [85,86,87,88,89,90,91]. If required, other approaches may be used to further enhance cellular immunity against HA, such as employing different prime–boost regimens, nanoparticle presentation, additional viral protein targets, or vectored strategies [57,92,93].

## 5. Conclusions

In summary, this work serves as a pilot study to demonstrate the ability of our vaccine strategy to induce effective humoral and cellular immune responses in the BALB/c model with different adjuvant–antigen pairings. We observed robust humoral immune responses for all adjuvants paired with HA clamp and for alhydrogel, AdQS21, and ALF55 groups paired with RSV F clamp. This study has highlighted the importance of adjuvant selection for different antigens, where the same adjuvant may elicit varying immune responses based on the antigen it is formulated with. We suggest that this could be in part due to different adsorption rates of antigen to adjuvant as well as intrinsic immune stimulatory effects of antigens alone that may work in synergism or hinder adjuvant effects. This is highlighted in the isotyping data, where AdQS21 elicited a well-rounded response for all isotypes when formulated with RSV F clamp; however, this was not reproduced in the HA clamp study. As such, we conclude that an adjuvant trial for different subunit vaccines in development would likely be necessary, with a necessity to conduct formulation work, particularly if the vaccine candidate was to progress into a challenge model or human clinical trials.

## Data Availability

The data presented in this study are available within the article and supplementary material.

## Supplementary Materials

The following are available online at https://www.mdpi.com/2076-393X/9/2/71/s1.
